# Reexpansion Pulmonary Edema following Tube Thoracostomy in a Pediatric Patient with Anterior Mediastinal Mass

**DOI:** 10.1155/2022/8547611

**Published:** 2022-05-19

**Authors:** Sung-Wook Choi, Deborah A. Romeo, David A. Gutman, Jennifer V. Smith

**Affiliations:** ^1^Anesthesia and Perioperative Medicine, Pediatric Anesthesia, Medical University of South Carolina, 10 McClennan Banks Drive, Suite 2190, MSC 940, Charleston, South Carolina 29425, USA; ^2^Anesthesia and Perioperative Medicine, Obstetric Anesthesia, Medical University of South Carolina, 10 McClennan Banks Drive, Suite 2190, MSC 940, Charleston, South Carolina 29425, USA

## Abstract

Reexpansion pulmonary edema (RPE) is an exceedingly rare and potentially fatal complication of a rapidly reexpanded lung following evacuation of air or fluid from the pleural space secondary to conditions such as a mediastinal mass, pleural effusion, or pneumothorax. Clinical presentations can range from mild radiographic changes to acute respiratory failure and hemodynamic instability. The rapidly progressive nature of the disease makes it important for clinicians to appropriately diagnose and manage patients who develop RPE. We present a case of a child with a large malignant pleural effusion who developed severe RPE after tube thoracostomy and ultimately required venoarterial extracorporeal membrane oxygenation (VA-ECMO). The patient was 7-year-old Caucasian male with newly diagnosed ambiguous T cell myeloid leukemia. A chest computerized tomography (CT) demonstrated a large pleural effusion causing tracheal shift and left bronchus compression as well as an anterior mediastinal mass causing compression of the right atria and right ventricle. Tube thoracostomy was performed in the operating room (OR) with deep sedation. The procedure was complicated with hypoxemia, bradycardia, and pulseless cardiac arrest. After return of spontaneous circulation, the child continued to have refractory hypoxemia, profound hypotension, and frothy secretions. Endotracheal intubation was performed with a size 5.0 cuffed endotracheal tube. Chest radiograph demonstrated opacification of the left hemithorax with chest infiltrates. Patient required VA-ECMO for circulatory support. Supportive therapy of RPE was continued and decannulation was done on day three. Tracheal extubation was performed on day five.

## 1. Introduction

Reexpansion pulmonary edema (RPE) is an exceptionally rare and potentially fatal complication of a rapidly reexpanded lung following evacuation of air or fluid from the pleural space. Clinical presentations can range from mild radiographic changes to acute respiratory failure and hemodynamic instability [1]. The rapidly progressive nature of the disease makes it important for clinicians to appropriately diagnose and manage patients who develop RPE. We present a case of a child with a large malignant pleural effusion and anterior mediastinal mass secondary to ambiguous T cell myeloid leukemia who developed RPE after tube thoracostomy. The patient was profoundly unstable and ultimately required venoarterial extracorporeal membrane oxygenation (VA-ECMO). Publication was approved by the patient's legal guardian as he is a minor. There is no identifiable information.

## 2. Case Presentation

### 2.1. Symptoms

**Figure 1:**
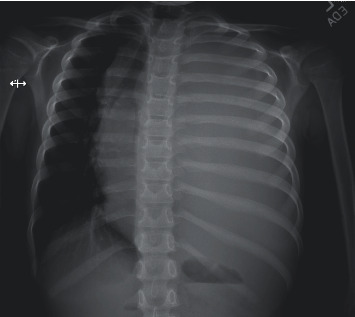
Chest radiograph with complete left-sided pleural effusion with a resultant mass effect and significant deviation of the mediastinum to the right.

We present the case of a previously healthy 7-year-old Caucasian male who presented to the hospital with 6 weeks of generalized malaise, weight loss, productive cough, and one week of lymphadenopathy with progressive dyspnea. The patient had normal vital signs, >92% SpO_2_, no accessory thoracic muscle use, and reported no respiratory distress. There was no postural variation of respiratory symptoms. On physical exam, the patient had absent breath sounds on the left and palpable, firm, and nontender lymph nodes in bilateral axillae. A chest radiograph on admission revealed complete opacification of the left hemithorax with a mass effect, including mediastinal shift to the right (Figure 1).

### 2.2. Diagnosis

**Figure 2:**
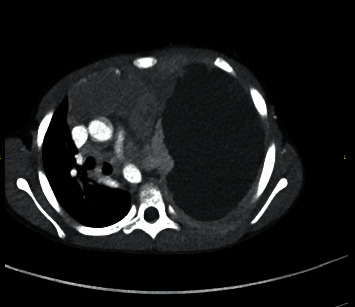
Chest computed tomography showing large right pleural effusion and anterior mediastinal mass with the mass effect.

Labs demonstrated a white blood cell count of 3.55 with 21% blasts and peripheral smear with atypical leukocytes, consistent with ambiguous lineage T cell myeloid leukemia. Chest computerized tomography (CT) was performed for further evaluation and revealed a large pleural effusion causing rightward shift of the trachea and compression with occlusion of the left lower bronchus, left upper bronchus, and superior lobe and lingual bronchi, as well as an anterior mediastinal mass measuring 8.5 × 4.0 × 6.0 cm with resultant right atrial and ventricular extrinsic compression (Figure 2). The patient was scheduled for tube thoracostomy placement in the operating room (OR). There was no indication for preprocedure arterial blood gas due to patient being conversant and in no respiratory distress.

### 2.3. Treatment

**Figure 3:**
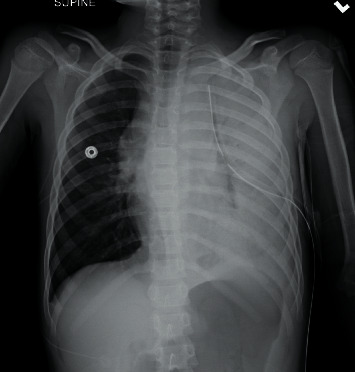
Immediate postchest tube thoracostomy chest radiograph demonstrating resolution of the mass effect and midline trachea.

The anesthesia goals in the OR were to avoid endotracheal intubation and positive pressure ventilation due to the patient's anterior mediastinal mass and potential for airway obstruction. The patient was placed on 2 liters via nasal cannula, and deep sedation was maintained with dexmedetomidine and remifentanil infusions supplemented with boluses of ketamine. Upon tube thoracostomy placement, 500 milliliters of serous pleural fluid were initially evacuated prior to clamping for 10 minutes. After release of the clamp, an additional 1400 milliliters of serous fluid were drained. Over the next 3 minutes, the patient progressively developed profound hypoxemia and bradycardia which culminated in cardiac arrest secondary to pulseless electrical activity. Cardiopulmonary resuscitation was initiated, and return of spontaneous circulation occurred after two minutes of chest compressions and one dose of epinephrine. An immediate postprocedure chest radiograph showed resolution of the mass effect and contralateral tracheal deviation, but persistent opacification of the left hemithorax (Figure 3).

### 2.4. Outcomes

**Figure 4:**
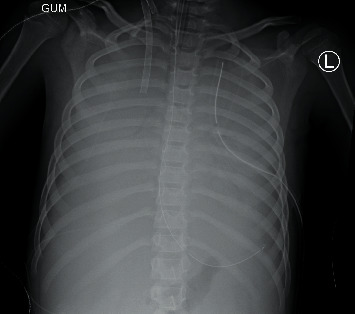
Chest radiograph showing progressive and complete opacification of bilateral lung fields.

After obtaining return of spontaneous circulation, the patient was transferred to the ICU for close monitoring. While there and approximately 45 minutes after the initial cardiac arrest, the patient developed refractory hypoxemia and hypotension and frothy secretions concerning for pulmonary edema. Airway was secured via rapid sequence intubation using etomidate and succinylcholine. It was a grade 1 view using a Miller 2 blade. Right femoral central venous and right radial arterial access were obtained. Epinephrine and vasopressin infusions were initiated for circulatory support. Due to continued refractory hypotension and serial chest radiographs concerning for progressively worsening lung opacification and chest infiltrates (Figure 4), the patient was placed on venoarterial extracorporeal membrane oxygenation (VA-ECMO).

**Figure 5:**
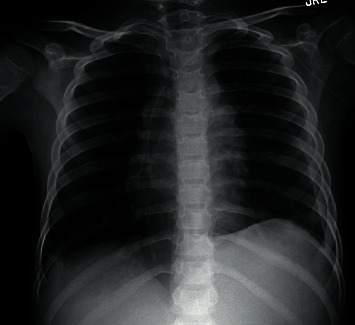
Chest radiograph before discharge demonstrating resolved pulmonary edema.

The patient received supportive care for RPE and was decannulated from ECMO on postprocedure day 3 and extubated on postprocedure day 5. Patient was discharged for outpatient follow-up on hospital day 26, with radiographic and clinical resolution of his RPE (Figure 5).

## 3. Discussion

RPE is an exceedingly rare and generally iatrogenic complication that occurs when a chronically collapsed lung is quickly reexpanded following evacuation of air or fluid from the pleural space. The incidence of RPE after intrapleural drainage has been cited as low as 1%, but mortality reported as high as 20% [2]. Symptomatology can range from benign nonspecific findings to respiratory failure with acute respiratory distress syndrome (ARDS)-like presentation, cardiovascular collapse, and death. In most cases, patients often exhibit symptoms within one hour of the inciting event [2]. This case of RPE is only the second pediatric patient described in the literature to have survived RPE requiring ECMO salvage therapy.

The pathophysiology of RPE is incompletely understood, but several proposed mechanisms involve the destruction of pulmonary capillary membranes via inflammation. This causes excess fluid permeation into the pulmonary interstitium and alveoli, resulting in diffuse pulmonary edema. Changes in membrane integrity due to hypoxemia, capillary wall injury, and decreased surfactant production can all be attributed to the chronically collapsed lung. Research also suggests that dramatic increases in hydrostatic pressure caused by a sudden increase in venous return can result in mechanical damage due to overstretching during reexpansion as well as fluid transudation across the injured capillaries and alveolar membranes. All these factors subsequently contribute to the release of inflammatory mediators such as interleukin-8 (IL-8), and monocyte chemotactic protein-1 (MCP-1), leukotriene B4 (LTB4), nitric oxide, and free radicals that in turn worsen the injury and further enhance capillary permeability. Of note, IL-8 and neutrophils are also found in high concentrations in other pathologic conditions such as ARDS and ischemia-reperfusion injuries. In one rabbit model study, investigators confirmed RPE was highly correlated with increased IL-8 and MCP-1 concentrations in bronchoalveolar lavage fluid [3]. They also interestingly noted that a similar but less severe inflammatory response was seen in the contralateral lung. This may partially explain how bilateral RPE can occur through a possible systemic inflammatory response, as seen in our patient.

Given that the cause of RPE is frequently noted to be iatrogenic in many reported cases, prevention strategies are paramount to ensuring the safety of any patient undergoing a procedure for large volume evacuation from the pleural space. Understanding the risk factors such as length of lung collapse (generally cited as greater than three days), amount of pleural air or fluid (greater than 1500 milliliters), application of negative pleural suction, rapidity of lung expansion, and preexisting hypoxemia or other lung disease can help clinicians guide treatment plans in cases similar to that described herein. Current literature advises clinicians to limit drainage to less than one liter of fluid or air in one sitting to decrease the risk of RPE. It is also preferable that drainage be performed with pleural pressure monitoring so as not to surpass 20 mmHg with frequent clamping of the thoracostomy tube [4].

Though rare, RPE should be considered when hemodynamic instability or cardiac collapse is encountered after intrapleural drainage. When significant, these symptoms can be refractory to circulatory support and endotracheal intubation and may require ECMO [5]. As symptoms can develop within 1–24 hours after intrapleural drainage, providers in the operating room as well as in intensive care units need to be able to quickly recognize and diagnose RPE as it can progress rapidly and progress to death if not treated immediately. Of even greater importance is to take preventative steps including slow and judicious removal to avoid the described complication.

## Data Availability

The data used to support this study are included within the article.
